# Fiber Memristor-Based Physical Reservoir Computing for Multimodal Sleep Monitoring

**DOI:** 10.34133/research.0870

**Published:** 2025-09-09

**Authors:** Jinhao Zhang, Zhenqian Zhu, Jialin Meng, Tianyu Wang

**Affiliations:** ^1^ School of Integrated Circuits, Shandong University, Jinan 250100, China.; ^2^ Suzhou Research Institute of Shandong University, Suzhou 215123, China.; ^3^ State Key Laboratory of Crystal Materials, Shandong University, Jinan 250100, China.; ^4^ National Integrated Circuit Innovation Center, Shanghai 201203, China.; ^5^ Key Laboratory of Computational Neuroscience and Brain-Inspired Intelligence (Fudan University), Ministry of Education, Shanghai 200433, China.

## Abstract

Real-time wearable sleep monitors process diverse biological signals while operating under tight energy and computation budgets. The existing algorithms are facing problems of high energy consumption due to separate hardware storage and computation units. In this work, textile-integrated in-memory neuromorphic computing electronics based on MoS_2_ quantum dot fiber memristors was proposed for physical reservoir computing for the first time. Textile electronics convert raw electroencephalogram (EEG)and snoring audio directly into rich, high-dimensional state vectors based on intrinsic nonlinear dynamics. Leveraging 16 pulse-programmable conductance levels, the reservoir realizes an accuracy of 94.8%, 95.4%, and 93.5% in snoring events, sleep stages, and multimodal fusion, respectively. To enhance the robustness of feature extraction and improve classification performance under noisy conditions, the linear readout layer was replaced with a lightweight convolutional neural network. The hybrid neural network is 6 times faster than traditional deep-learning methods in 24-h segment EEG analysis. The memristors switch at ±1 V and sub-nanoampere currents, providing picowatt energy consumption suited to continuous on-body use. The results establish fiber memristor reservoir computing as an energy-efficient path to in-fabric, multimodal intelligence for next-generation home sleep analysis and wearable health care.

## Introduction

Wearable health monitoring systems are increasingly expected to analyze diverse real-time biological signals, offering actionable insight into physiological states such as sleep quality [1–4]. However, this task presents substantial computational and practical challenges [5,6]. Sleep monitoring requires the joint interpretation of multimodal signals such as electroencephalogram (EEG) and snoring sounds, which are often captured under noisy unconstrained environments [7–9]. EEG reflects dynamic brain activity over time, while snoring audio provides acoustic markers linked to sleep-disordered breathing [10–12]. These signals differ in temporal structure, frequency content, and dimensionality, making real-time fusion and classification difficult in resource-constrained environments [13]. Some methods have been developed to deal with biological signals, such as convolutional neural network (CNN)-based audio classifiers, recurrent neural network (RNN)-based EEG analysis systems, and transformer models. Traditional machine learning pipelines, typically deployed on von Neumann architectures, face high memory traffic and energy demands [14–16]. Separate memory and processing units result in frequent data shuttling and lead to excessive memory traffic and energy inefficiencies, especially for time-series tasks requiring long sequence retention or frequent input and output. These limitations highlight the need for energy-efficient, fabric-integrated neuromorphic computing methods capable of real-time multimodal inference [17–19].

Memristors offer a low-power alternative by combining data storage and computation in a single compact device and excluding costly memory access [20,21]. Among recent implementations of physical reservoir computing (RC), a range of device platforms have been explored, including oxide memristors, phase-change materials, and optoelectronic systems [22–25], as well as magnetic skyrmions, transistor-based architectures, and photonic RC devices that have demonstrated promising dynamics for temporal signal processing [26–30]. Recent studies have demonstrated in-sensor and dual-mode RC architectures using optoelectronic and ferroelectric memristors, which reveal substantial promise for low-power edge artificial intelligence (AI) in health and sensory tasks [31–33]. However, these architectures are often rigid, planar, or reliant on external digital modules, making them poorly suited for continuous, on-body use. Fiber-shaped memristors uniquely satisfy the demands of wearable health monitoring by coupling mechanical flexibility with pulse-programmable nonlinear dynamics [34–37]. Their time-dependent conductance modulation naturally maps onto RC principles, allowing temporal biological signals to be transformed into high-dimensional state behavior with minimal training overhead [38,39]. Therefore, the fiber memristor represents a promising path toward real-time, multimodal inference for physical RC.

In this work, we present a textile-integrated neuromorphic platform in which a MoS_2_ quantum dot (QD) fiber memristor performs physical RC for real-time multimodal sleep monitoring. The memristive physical reservoir converts both EEG waveforms and snoring acoustics into analog current behavior across 16 pulse-programmable conductance states. Leveraging these intrinsic dynamics, the system classifies sleep stages with an accuracy of 95.4%, detects snoring events at 94.8%, and attains 93.5% accuracy in multimodal fusion tasks. The reservoir operates at sub-nanoampere currents under ±1 V biases, yielding ultralow-power consumption compatible with overnight wearable use. These results establish fiber memristor reservoirs as an energy-efficient route to fabric-embedded, multimodal inference for continuous health monitoring in real-world settings.

## Results and Discussion

**Fig. 1.**
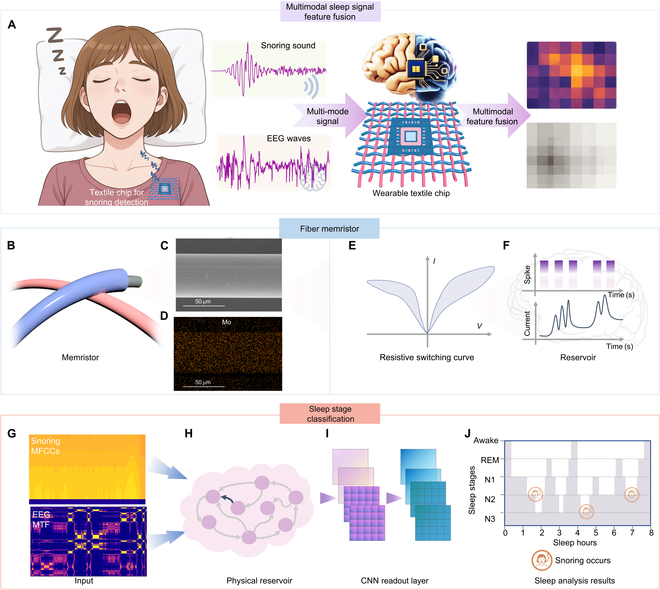
Fiber memristor-based physical reservoir computing for multimodal sleep monitoring. (A) Schematic of multimodal biological signal fusion using the fiber memristor, combining snoring acoustics and EEG signals for multimodal sleep monitoring. (B) Illustration of the coaxial architecture of the Ag/MoS_2_ QDs/Ag fiber memristor cross-point. (C) Scanning electron microscopy (SEM) image of the fiber surface structure. (D) Elemental mapping of molybdenum (Mo) confirming uniform QD distribution. (E) Representative bipolar resistive switching (RS) curve demonstrating memristive behavior. (F) Schematic of the dynamic current response generated in the physical reservoir. (G) Input signal representations: Mel-frequency cepstral coefficients (MFCCs) for snoring and a Markov transition field (MTF) matrix for EEG. (H) Illustration of the fiber memristor-based reservoir. (I) The CNN readout layer used for sleep monitoring training, replacing the linear layer to enhance feature extraction from reservoir states. (J) Final sleep monitoring timeline result across the full night based on multimodal classification.

To achieve continuous wearable sleep monitoring across diverse multimodal biological signals, we designed a textile-integrated physical RC system centered on a MoS_2_ QD-based fiber memristor. As shown in Fig. 1A, the platform directly processes snoring and EEG signals by projecting them into a shared high-dimensional state space through nonlinear dynamics. This integration enables real-time inference on fused modalities. The core device comprises a coaxial Ag/MoS_2_ QD/Ag architecture (Fig. 1B), fabricated directly onto a flexible fiber substrate. Structural and compositional characterization confirmed the uniform coating of MoS_2_ QDs around the conductive fiber core. SEM imaging (Fig. 1C) reveals the fibrous topography of the surface, while elemental mapping of Mo (Fig. 1D) verifies uniform QD distribution along the device cross-section. This conformal structure is critical for signal stability under mechanical deformation. To validate its neuromorphic switching behavior, we measured the device’s resistive switching response under pulse stimuli. The resistive switching *I*–*V* curve in Fig. 1E demonstrates robust conductance modulation, forming the basis of its reservoir dynamics. As illustrated in Fig. 1F, each input pulse triggers a nonlinear current evolution, enabling temporal encoding via a fixed bank of 16 discrete conductance states. These transitions are exploited to encode biological signals into continuous time-dependent behavior.

Biological signal preprocessing transforms raw EEG and snoring data into compact yet informative representations: EEG is mapped into a Markov transition field (MTF), while snoring audio is represented via mel-frequency cepstral coefficients (MFCCs) as shown in Fig. 1G. These formats preserve temporal dependencies and spectrum features essential for classification. The memristor reservoir (Fig. 1H) then projects these signals into a high-dimensional response space. To enhance the model’s ability to learn temporal and spatial signal dependencies, we replaced the conventional linear readout layer with a simplified CNN structure (Fig. 1I). This modification allows for local feature extraction across memristor response states while keeping the computational footprint minimal. Across an overnight test, the system successfully performed multimodal monitoring using real EEG and snoring inputs. The final classification timeline (Fig. 1J) demonstrates accurate sleep stages classification and snoring events detection, laying the foundation for fully integrated low-power fiber memristor for sleep analysis.

**Fig. 2.**
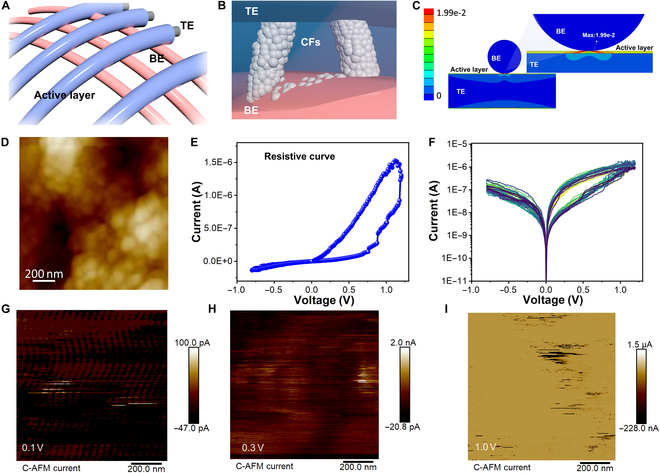
(A) Schematic of the fiber memristor. (B) Schematic of conductive filaments in the “on” state, showing conductive pathways within the memristor. (C) Simulation of force distribution at the intersection. (D) Atomic force microscopy (AFM) image of the quantum dot layer, providing surface morphology details. (E) Typical resistive curve *I*–*V* characteristic of the Ag/MoS_2_ QDs fiber memristor, showing clear bipolar resistive switching behavior with an ON/OFF current ratio. (F) Log-scale *I*–*V* curves measured over 20 consecutive cycles, demonstrating highly stable switching performance and excellent cycle-to-cycle reproducibility. C-AFM current map at the increasing dynamic ranges: (G) pA scale, illustrating the initial formation of conductive filaments; (H) nA scale showing the growth and increased conductivity of the filaments; and (I) μA scale depicting the fully developed conductive pathways.

The structure of the Ag/MoS_2_ QDs/Ag fiber memristor is the core computing element in our physical RC (Fig. 2A and Fig. S1). This structure integrates flexibility and conductivity, making it suitable for continuous on-body applications. The MoS_2_ QDs layer enables controllable resistive switching, essential for simulating the nonlinear, memory behavior of biological synapses. Under external bias, conductive filaments grow through the QD layer (Fig. 2B and Fig. S2). To assess mechanical robustness under real-use conditions, we conducted finite element simulations of stress distribution at the electrode intersection (Fig. 2C and Fig. S3). The coaxial fiber geometry evenly distributes mechanical strain during bending and stretching, supporting reliable operation in dynamic wearable environments.

Surface topography of the QD switching layer was characterized by atomic force microscopy (AFM) as shown in Fig. 2D. The uniform nanoscale granular texture is beneficial for local electric field enhancement and ion mobility. The memristor now demonstrates robust bipolar resistive switching behavior as shown in Fig. 2E. The clear low-resistance state (LRS) and high-resistance state (HRS) enable robust state separation suitable for neuromorphic synaptic weighting. To validate switching stability, we conducted 20 cycles *I*–*V* measurements (Fig. 2F), which reveal highly repeatable hysteresis loops with negligible cycle-to-cycle variation, confirming reliable filament formation and rupture dynamics over extended operation. These enhancements substantially strengthen the device’s suitability for neuromorphic and RC applications. To further probe the switching mechanism at the nanoscale, we performed conductive AFM (C-AFM) imaging at increasing current detection ranges. The C-AFM mapping of the gradual formation of conductive filaments was tested under increasing bias voltages of 0.1, 0.3, and 1.0 V, respectively. At the picoampere level (Fig. 2G), isolated nucleation sites of conductive filaments appear. As the readout range increases to the nanoampere scale (Fig. 2H), these filaments grow and begin to form extended pathways. Finally, at the microampere scale (Fig. 2I), a continuous conduction network emerges—corresponding to the full formation of an LRS. These observations are consistent with an electrochemical metallization mechanism, in which Ag^+^ ions from the active electrode migrate through the MoS₂ QD switching layer under the influence of an electric field and are subsequently reduced to form metallic filaments. The QD morphology and the presence of chalcogen vacancies in MoS₂ enhance ion mobility and facilitate localized field concentration, thereby accelerating filament nucleation. Upon reversal of the bias, the conductive filaments dissolve, restoring the HRS. This dynamic and reversible filament evolution underlies the device’s nonvolatile memory characteristics and mimics short-term to long-term plasticity observed in biological synapses—making it particularly suitable for neuromorphic and RC applications. X-ray photoelectron spectroscopy was performed to examine the chemical bonding state of the MoS₂ QD layer, indicating the successful formation of stoichiometric MoS₂ QDs without substantial oxidation or contamination (Fig. S4). This confirms the high purity and chemical integrity of the active material, essential for stable resistive switching behavior. These results confirm that the Ag/MoS_2_ QD/Ag fiber memristor exhibits robust, tunable, and spatially localized resistive switching with excellent mechanical resilience. These properties establish the device as a promising candidate for signal processing in flexible, low-power neuromorphic systems.

**Fig. 3.**
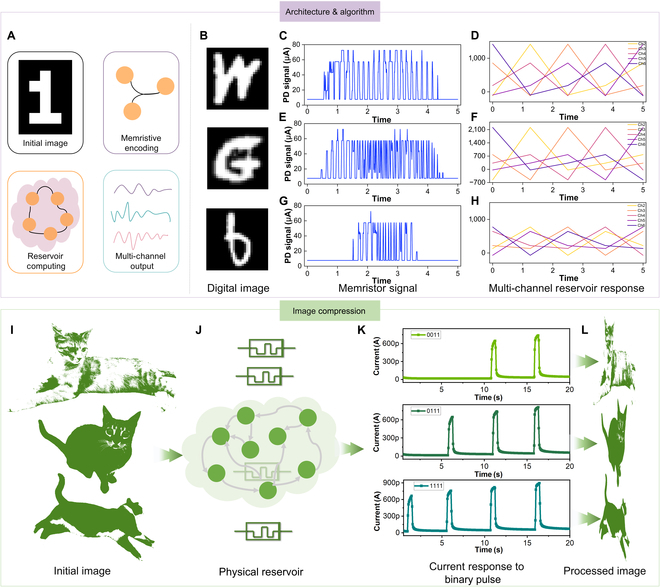
Memristor-based physical reservoir computing for image signal transformation and compression. (A) Schematic illustration of image compression using memristor-enabled physical reservoir computing process. (B) Three handwritten digit images (“W”, “G”, and “6”) from the MNIST dataset. (C to H) Memristor signal response to the handwritten input image “W”, “G”, and “6”. (I) Three binarized real-world cat images used as compression input. (J) Schematic of the memristor-based physical reservoir used for image compression. (K) Current responses to binary pulse inputs representing different pixel states, showing the memristor’s analog transformation capability. (L) Final compressed image output reconstructed from reservoir states, demonstrating preservation of structural features after compression.

To evaluate the generalization capability of our fiber memristor RC architecture, we applied it to a typical image compression task. This experiment demonstrates the platform’s versatility in handling spatially encoded information using the same pulse-driven memristor. Figure 3A presents the conceptual pipeline for memristor-based image signal compression. Input images are encoded into pulse sequences according to pixel intensity and fed into the memristor. The device’s current response captures the temporal and spatial information embedded in the input, transforming it into a compact representation. We selected 3 handwritten symbols (“W”, “G”, and “6”) from the Modified National Institute of Standards and Technology(MNIST) dataset as representative input patterns (Fig. 3B). Each pixel with intensity above a set threshold was encoded into a fixed-voltage pulse (1.5 V, 200 ms), while zero-value pixels remained unstimulated. These pulses were delivered to spatially defined test points along the fiber device, enabling localized modulation of the memristor conductance. Figure 3C, E, and G show the memristor current responses corresponding to symbols “W”, “G”, and “6”, respectively. The distinct current profiles reflect the spatial arrangement and density of activated pixels. The resulting multi-channel reservoir outputs (Fig. 3D, F, and H) exhibit clear differentiation in conductance states across the device, effectively mapping visual structure into a high-dimensional analog response. The memristor preserves distinguishable signal patterns for each input, underscoring its analog selectivity and encoding capacity.

To further validate applicability to real-world visual input, we used 3 binarized cat images as test inputs (Fig. 3I). The physical RC structure (Fig. 3J) remained unchanged, and the different pulse-to-current transformations (Fig. 3K) were used to generate compressed analog outputs. The final compressed images reconstructed from reservoir outputs (Fig. 3L) retain critical edge and shape features, demonstrating effective compression while maintaining semantic integrity. This result highlights the ability of our fiber memristor-based physical reservoir to encode spatial features across diverse input modalities. The Ag/MoS_2_/Ag memristors exhibit faster dynamic switching speeds than the 1- to 10-ms synaptic delays typically found in biological systems, enabling real-time response and low-latency processing. This rapid switching supports the fast encoding of visual information through pulse-controlled conductance modulation, positioning our architecture as a viable platform for general-purpose signal processing in flexible, neuromorphic fabrics. The low-power pulse-controlled conductance modulation transformation opens a pathway to general signal processing in flexible neuromorphic fabrics.

**Fig. 4.**
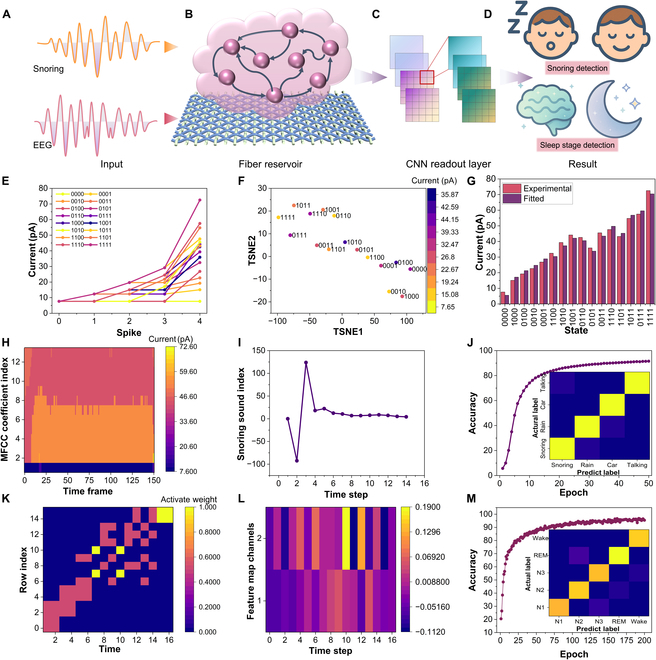
Separate processing and classification of snoring and EEG signals using fiber memristor-based physical reservoir computing. (A to D) Schematic of the snoring and EEG signal processing pipeline: (A) multimodal inputs consisting of acoustic snoring and EEG signals; (B) temporal transformation via the fiber memristor-based physical reservoir; (C) a light CNN readout layer for classification; (D) conceptual illustration of final recognition tasks including snoring detection and sleep stage classification. (E) Sixteen pulse-programmable conductance states in memristor. (F) t-SNE visualization of memristor responses, highlighting nonlinear state evolution on cluster separability. (G) Bar graph comparing experimentally measured memristor currents with model-fitted values, showing accurate modeling of the memristor’s nonlinear response. (H) Processed high-dimensional snoring MFCC spectrogram by the memristor. (I) High-dimensional response to snoring in memristor. (J) Accuracy and confusion matrix for snoring detection. (K) Processed high-dimensional EEG MTF signal. (L) Class activation map (CAM) from the linear readout layer showing activation regions to EEG signals. (M) Classification accuracy and confusion matrix for EEG-based sleep stage recognition.

To validate the signal separation and classification capabilities of the fiber memristor-based reservoir, we conducted evaluations on snoring acoustics and EEG signals independently [40,41]. The full pipeline for each modality is illustrated in Fig. 4A to D, including preprocessing, analog transformation via the fiber reservoir, and a light CNN readout layer for final classification. The core of the system relies on the memristor’s 16 pulse-programmable conductance states (Fig. 4E and Table S1), which encode the temporal structure of incoming signals into current responses with all devices initialized from the same baseline current. These nonlinear transitions allow for complex feature mapping within a compact physical fiber reservoir. While 16 discrete levels are sufficient for the classification tasks demonstrated, they remain less granular than biological synapses, which can exhibit over 100 distinguishable weights. This limitation arises mainly from variability in filament growth and partial overlap between adjacent conductance levels. Future improvements may involve pulse modulation refinement or material doping to enhance resolution. To visualize the dynamic separation of input signals, we employed t-distributed stochastic neighbor embedding (t-SNE) analysis of the memristor response space (Fig. 4F). The resulting plot exposes well-separated clusters, confirming that distinct pulse sequences lead to unique and reproducible analog states—a hallmark of effective physical reservoir behavior. The nonlinear response of the memristor was further validated by comparing experimentally measured current outputs with the modeled values used in the reservoir simulation as shown in Fig. 4G. The close consistency between experimental and fitted data demonstrates high precision in analog signal encoding and model integration.

Snoring signals were preprocessed using MFCCs to preserve spectral features, and then high-dimensional features were mapped by memristor as shown in Fig. 4H and Figs. S5 to S7. The resulting current responses (Fig. 4I) display clear snoring events. Our physical RC achieves accuracy at 94.8% on snoring detection with performance metrics and confusion matrix in Fig. 4J. For EEG signals, the MTF was mapped into high-dimensional structured matrices by memristor (Fig. 4K and Fig. S8). The memristor reservoir effectively transformed these matrices into nonlinear analog states, allowing the trained classifier to distinguish between sleep stages. The class activation map (CAM) highlights the regions in the signal that most influenced the network’s decisions (Fig. 4L and Fig. S9). Overall classification performance is shown in Fig. 4M, with an accuracy of 95.4% and clear separability across wake, light, and deep sleep stages. These results confirm that the fiber memristor reservoir can independently process and accurately classify diverse biological signals using low-power, analog dynamics. It provides a foundational step toward real-time, multimodal sleep analytics.

**Fig. 5.**
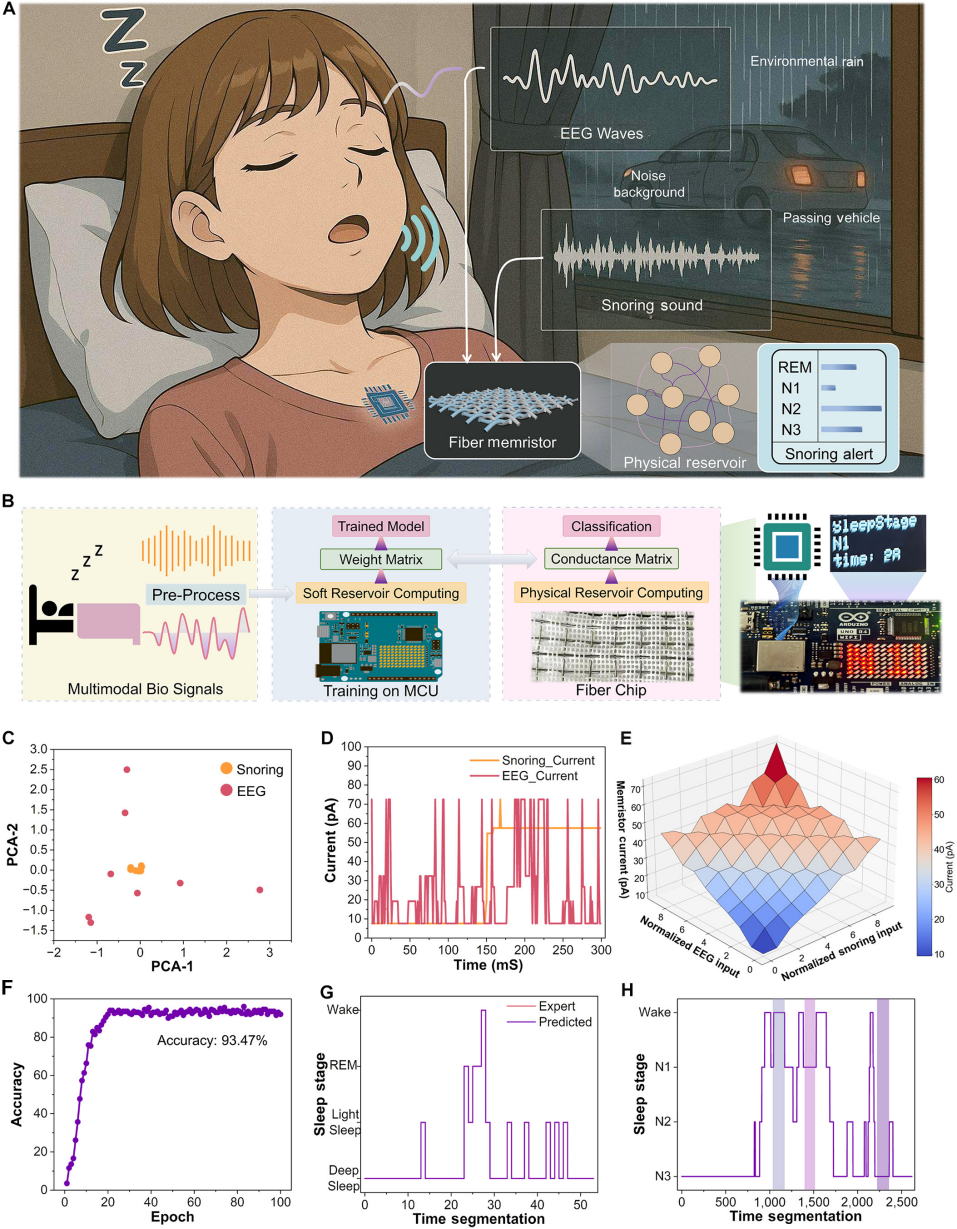
Multimodal validation of fiber memristor-based sleep monitoring system. (A) Illustration of the multimodal testing scenario, simulating real-world overnight sleep with concurrent snoring, environmental rainfall, and vehicular background noise. (B) The process of overnight multimodal sleep detection by fiber memristor. (C) Principal component analysis (PCA) to EEG and snoring responses by memristor, demonstrating successful separation of the 2 modalities in feature space. (D) Current output of the memristor under both EEG and snoring input, showing distinct dynamic responses. (E) Three-dimensional current surface under dual-modal stimulation, illustrating how the physical reservoir encodes complex temporal features. (F) Classification accuracy of the multimodal system during sleep monitoring tasks, validating robust inference performance in realistic conditions. (G) Sleep stage classification output compared with ground-truth annotations from human experts. (H) Twenty-four-hour sleep monitoring results, color-coded to indicate predicted states of wakefulness, light sleep, and deep sleep.

To demonstrate the practical relevance and robustness of our system, we conducted multimodal sleep monitoring experiments in a simulated real-world environment. As shown in Fig. 5A, the scenario includes continuous EEG and snoring inputs accompanied by rainfall and traffic background noise. We build a challenging condition for wearable health monitoring platforms (Fig. S10). Multimodal biological signals are first preprocessed and input into a microcontroller unit, where a soft RC algorithm is executed on the graphics processing unit to train a neural network model. During training, the system learns to map high-dimensional temporal features to output classes (e.g., sleep stages or snoring events), and a corresponding readout weight matrix is generated. These weights are then quantized and translated into discrete conductance values, which are mapped onto the physical memristor array. In this way, the learned model is embedded into the device’s analog hardware through the tuning of memristor conductance states. Final classification and inference are performed directly on the fiber-based physical RC chip, enabling real-time, on-device multimodal analysis (Fig. 5B and Figs. S11 and S12). The memristor’s ability to separate and encode concurrent multimodal signals was first evaluated. Principal component analysis (PCA) applied to the reservoir responses revealed clear clustering of EEG and snoring signals (Fig. 5C). The physical reservoir retains a clear and distinguishable encoding capacity even when signals from two modalities are input simultaneously. The temporal current output from the fiber memristor (Fig. 5D) exhibited different structured behavior for the 2 modalities, supporting dynamic encoding without interference. To further explore the encoding behavior under dual-modal conditions, we visualized the current surface in 3 dimensions (Fig. 5E and Fig. S13). The surface demonstrates a smooth but non-linear projection of joint EEG-snoring input into the memristor’s analog state space, illustrating how temporal features from both channels are embedded into a unified reservoir response.

Multimodal classification accuracy under these realistic conditions reached 93.5% (Fig. 5F and Figs. S14 to S16), highlighting the fiber reservoir’s ability to maintain performance under environmental noise and signal overlap. The predicted sleep stage outputs closely tracked human-expert annotations (Fig. 5G and Fig. S17), verifying the clinical validity of the classification across multiple stages. We further deployed the system in a continuous 24-h sleep tracking experiment (Fig. 5H and Figs. S18 and S19). The fiber-based reservoir consistently captured transitions between wakefulness, light sleep, and deep sleep, color-coded in the output timeline. These results demonstrate the potential of our system for long-duration, real-world deployment in wearable health devices. The findings confirm that the fiber memristor-based physical reservoir supports reliable, energy-efficient multimodal inference in real time, even under noisy and variable input conditions—validating its potential as a fabric-integrated intelligence layer for next-generation sleep analytics.

## Conclusion

In summary, we report a physical reservoir neuromorphic computing platform for real-time, multimodal sleep monitoring. The novel fiber memristor with the structure of Ag/MoS_2_ QDs/Ag was fabricated as core elements of a physical reservoir. Finite element simulations confirm the mechanical robustness of the coaxial architecture under stress, supporting its suitability for long-term wearable use. The fiber reservoir converts both EEG waveforms and snoring acoustics into high-dimensional analog behavior by intrinsic dynamics. The system achieves snoring detection and sleep stage classification accuracies of 94.8% and 95.4%, respectively, and reaches 93.5% accuracy in multimodal fusion tasks. With sub-nanoampere operating currents under ±1 V bias, the platform consumes orders of magnitude less power than conventional digital pipelines, making it viable for continuous overnight operation. Compared to conventional oxide-based memristors, phase-change materials, or optical RC systems, our approach offers unique advantages in flexibility, low-voltage operation, and seamless integration into textile-based electronics. However, challenges such as scalable fabrication, variability control, and integration density remain. Despite these limitations, our study demonstrates that combining fiber-shaped neuromorphic devices with efficient CNN-based readout layers can enable robust, adaptive, and energy-efficient health monitoring in real-world, wearable contexts. The framework is readily extendable to additional biosignals such as electrocardiogram, electromyogram, or ambient audio, laying the foundation for future multimodal, on-body AI systems.

## Materials and Methods

### Fabrication of fiber memristor

MoS_2_ QDs were synthesized and dispersed in a solution at a concentration of 1 mg/ml. Ag fiber was immersed in this QD solution and polarized under 40 V for 3 min. The resulting Ag/MoS_2_ fiber was then dried at room temperature to remove any residual solvent. Ag fibers were carefully interwoven with the Ag/MoS_2_ fiber to form the textile memristor.

### Device characterizations

AFM (Bruker Dimension Icon) was used to analyze the film morphology. For electrical measurements, the Ag/MoS_2_ fibers were connected to an Agilent B1500A semiconductor parameter analyzer to supply bias, while the Ag fiber was grounded.

### Multimodal feature fusion method

EEG signals were transformed using MTFs, and snoring audio was encoded via MFCCs. The extracted features were sequentially input into the memristor reservoir for dynamic state mapping. The reservoir responses from both modalities were concatenated to form a unified feature vector, which was fed into a CNN readout for classification.

## Data Availability

The data that support the findings of this study are available from the corresponding authors upon reasonable request.
