# Supplementary material for: Fiber Memristor-Based Physical Reservoir Computing for Multimodal Sleep Monitoring
Source: Research (Wash D C). 2025 Sep 9;8:0870. doi: 10.34133/research.0870 (PMC12417633; doi:10.34133/research.0870)
Supplement: Supplementary 1 — Figs. S1 to S19 Table S1 [file research.0870.f1.docx]

**Supporting Information**

**Fiber Memristor-Based Physical Reservoir Computing for Multimodal Sleep Monitoring**

*Jinhao Zhang^1^, Zhenqian Zhu^1^, Jialin Meng^1,2,3,4*^, and Tianyu Wang^1,2,3,4*^*

*^1^**School of Integrated Circuits, Shandong University, Jinan 250100, China; Suzhou Research Institute of Shandong University, Suzhou, 215123, China;*

*^2^State Key Laboratory of Crystal Materials, Shandong University, Jinan, 250100, China*

*^3^National Integrated Circuit Innovation Center, Shanghai 201203, China;*

*^4^Key Laboratory of Computational Neuroscience and Brain-Inspired Intelligence (Fudan University), Ministry of Education, Shanghai 200433, P. R. China*

**Email: jlmeng@sdu.edu.cn;*[*tywang@sdu.edu.cn*](mailto:tywang@sdu.edu.cn)

**The supporting information file includes:**

Figure S1. Schematic of fabrication process of fiber memristor.

Figure S2. The transition between high- and low-resistance states.

Figure S3. Force simulation when the load gradually increases.

Figure S4.X-ray Photoelectron Spectroscopy (XPS) of the MoS₂ QDs layer.

Figure S5. The obvious snoring sound in the 10s audio.

Figure S6. The MFCCs for Snoring

Figure S7. The MFCCs for non-Snoring

Figure S8. The Analysis for Choosing Markov Transition Field for EEG

Figure S9. Class Activation Map of all 5 classes through the physical reservoir computing.

Figure S10. PCA for Snoring and non-Snoring in noisy background

Figure S11. Robustness of multimodal classification under environmental noise.

Figure S12.The Multimodal sleep detection result for different sleep stages with snoring.

Figure S13. t-SNE analysis for EEG and Soring signals

Figure S14. Accuracy of 3 scales for Physical Reservoir Computing.

Figure S15. Loss of 3 scales for Physical Reservoir Computing.

Figure S16. Confusion Matrix for Physical Reservoir Computing.

Figure S17. Hypnogram of True and Predicted Sleep Stages Results.

Figure S18. EEG Z-Ratio Over 24 Hours by physical reservoir computing.

Figure S19. Detected Sleep Stages Over 24 Hours by physical reservoir computing

Table S1. Initial Experimental Final Current.


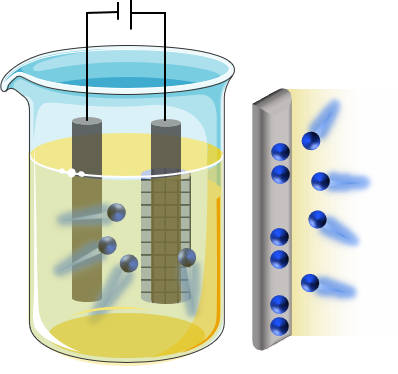


**Figure S1.** Schematic of fabrication process of fiber memristor through Electric-Field-Assisted assembly.


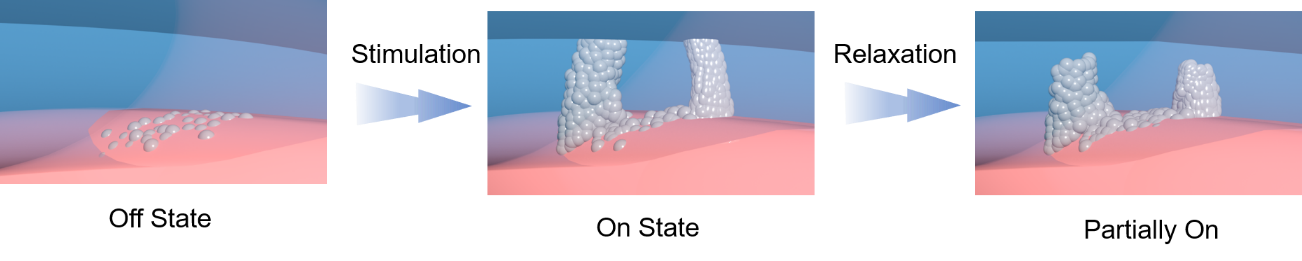


**Figure S2.** The transition between high- and low-resistance states.


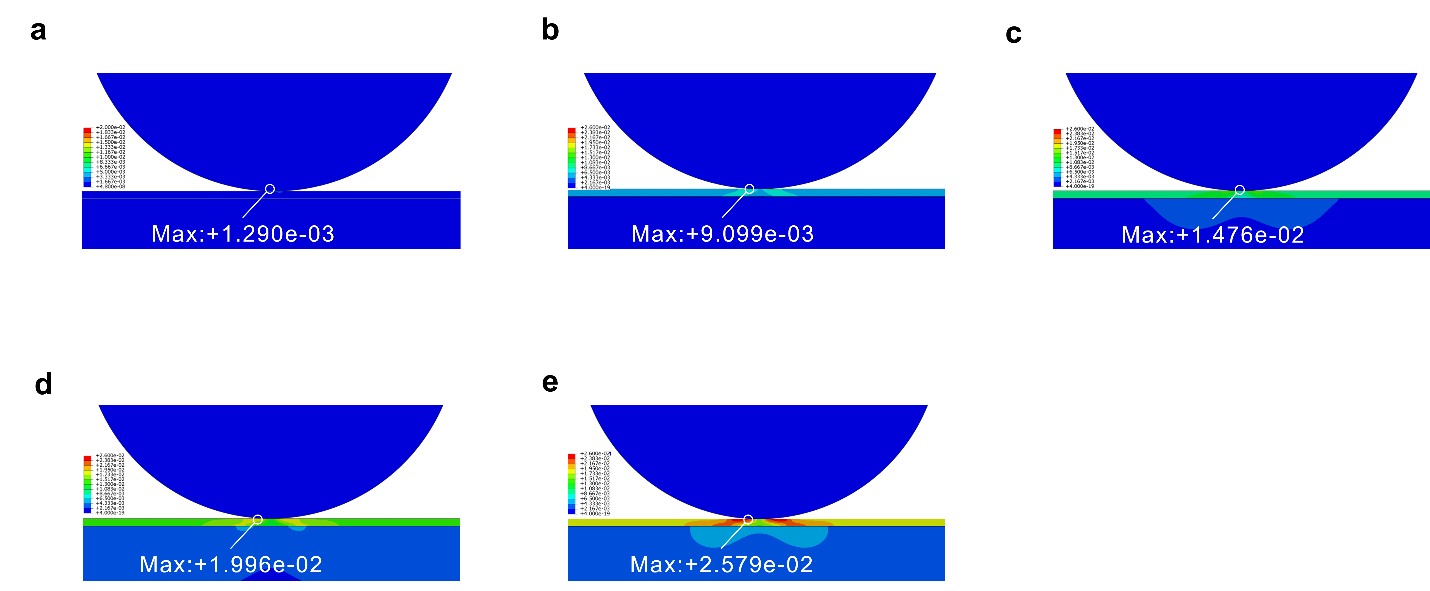


**Figure S3.** Force simulation when the load gradually increases. (a-e) Load increases at 2μm, 9μm, 16μm, 23μm, 30μm,


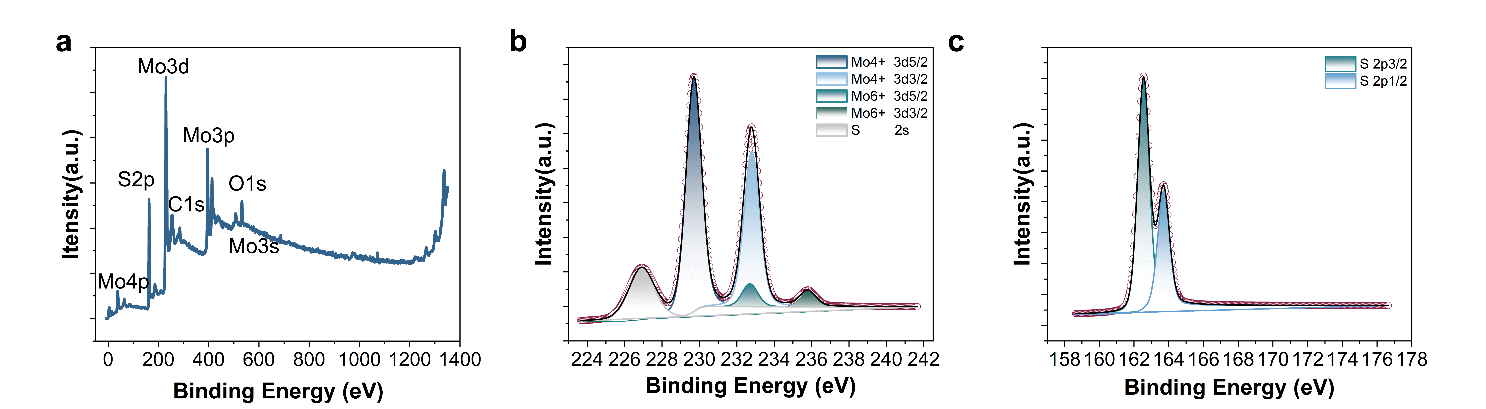


**Figure S4.**X-ray Photoelectron Spectroscopy (XPS) of the MoS₂ QDs layer.

X-ray Photoelectron Spectroscopy (XPS) was performed to examine the chemical bonding state of the MoS₂ QD layer. The Mo 3d and S 2p spectra reveal the characteristic doublet peaks corresponding to Mo⁴⁺ 3d₅/₂ and Mo⁴⁺ 3d₃/₂, as well as S²⁻ 2p₃/₂ and S²⁻ 2p₁/₂, indicating the successful formation of stoichiometric MoS₂ QDs without significant oxidation or contamination. This confirms the high purity and chemical integrity of the active material, essential for stable resistive switching behavior.


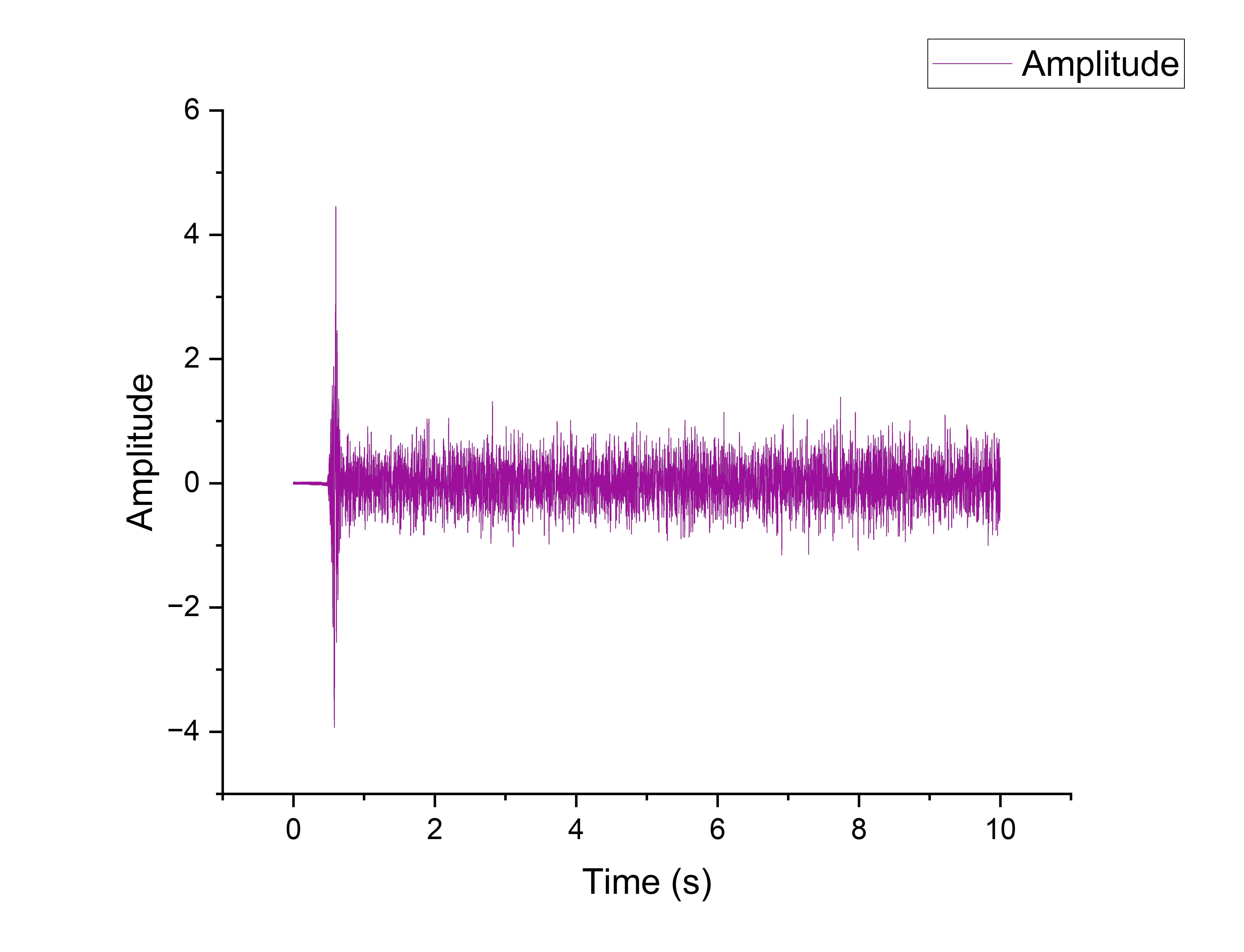


**Figure S5.** The obvious snoring sound in the 10s audio.


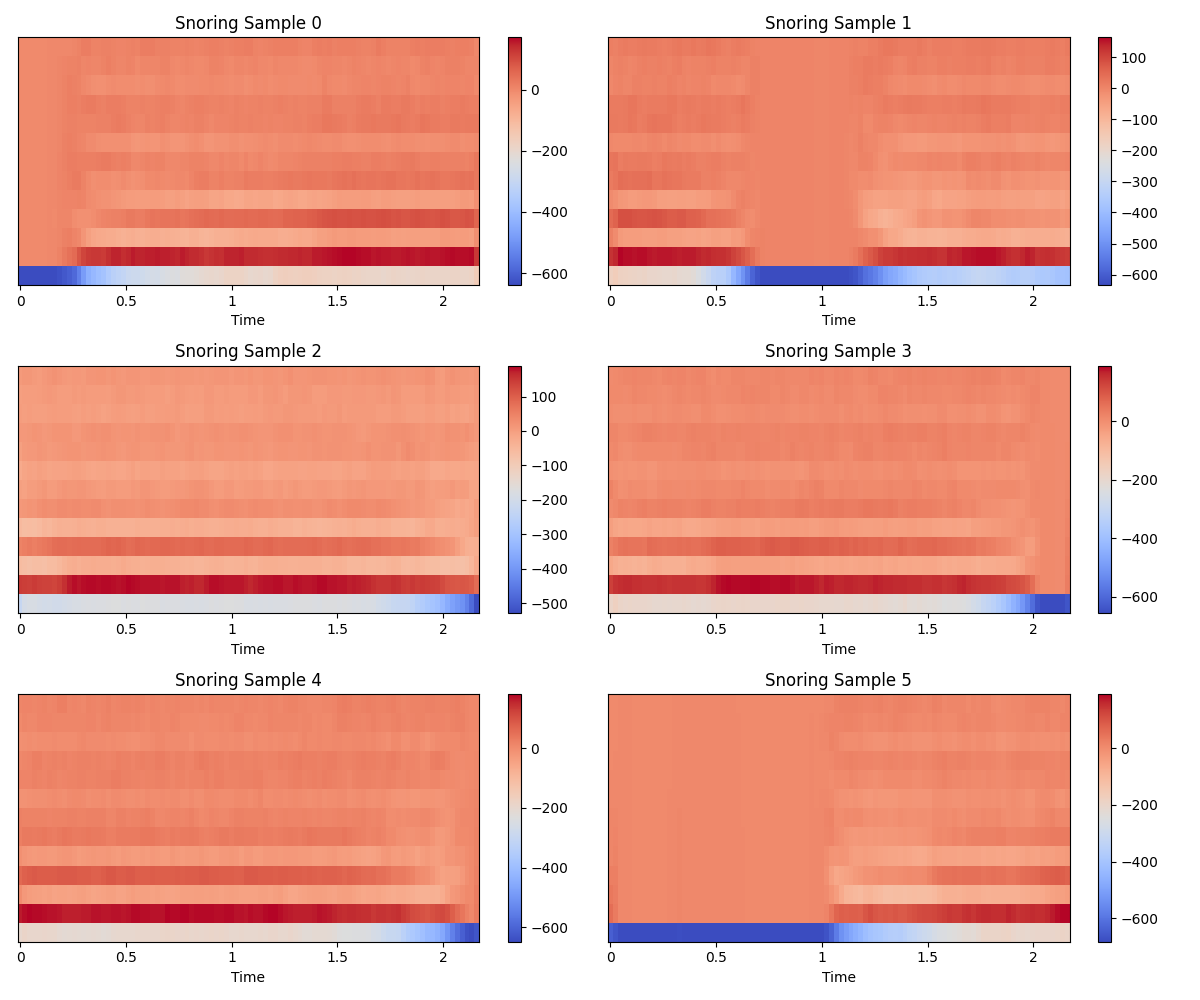


**Figure S6**. The MFCCs for Snoring


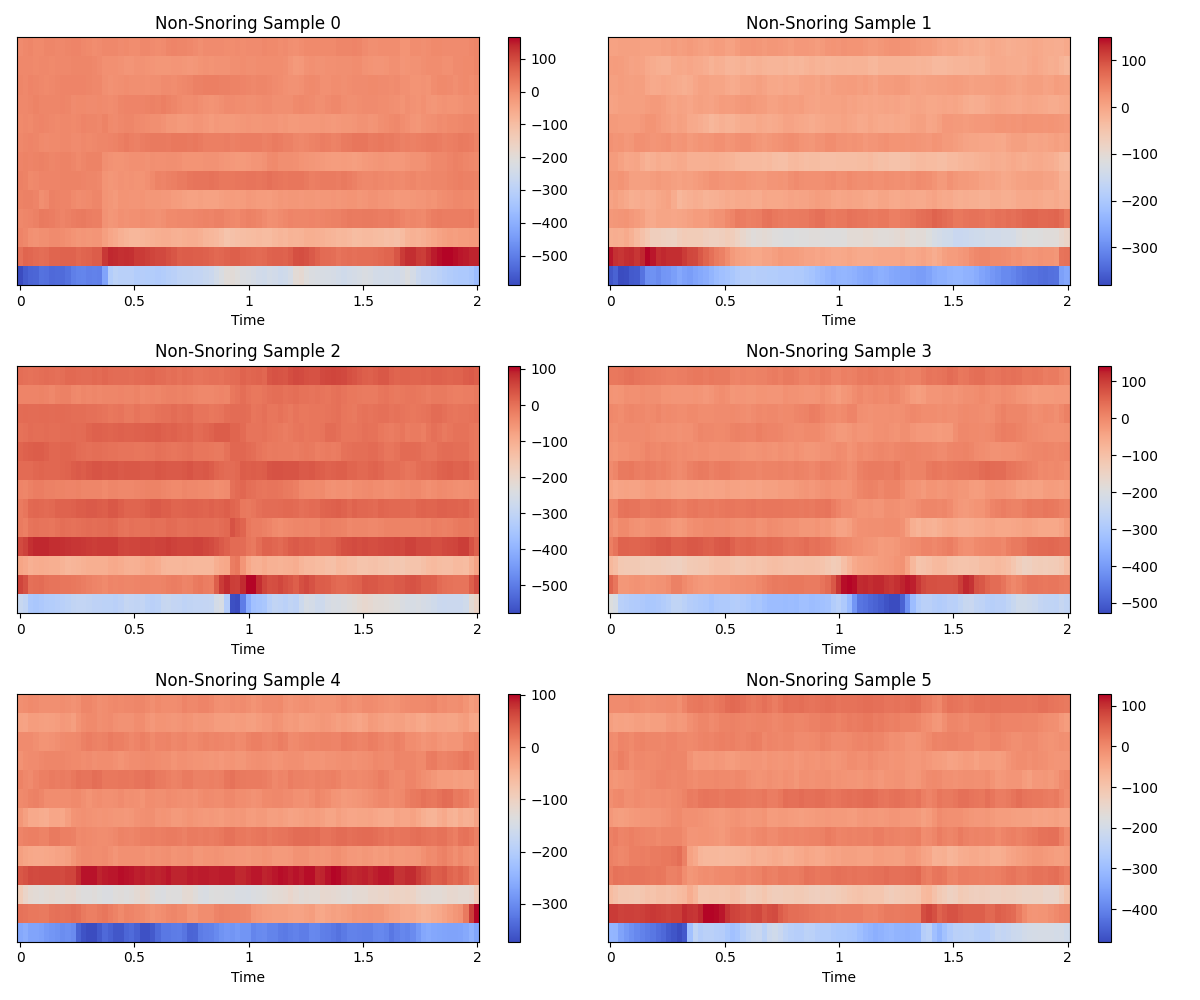
**Figure S7.** The MFCCs for non-Snoring


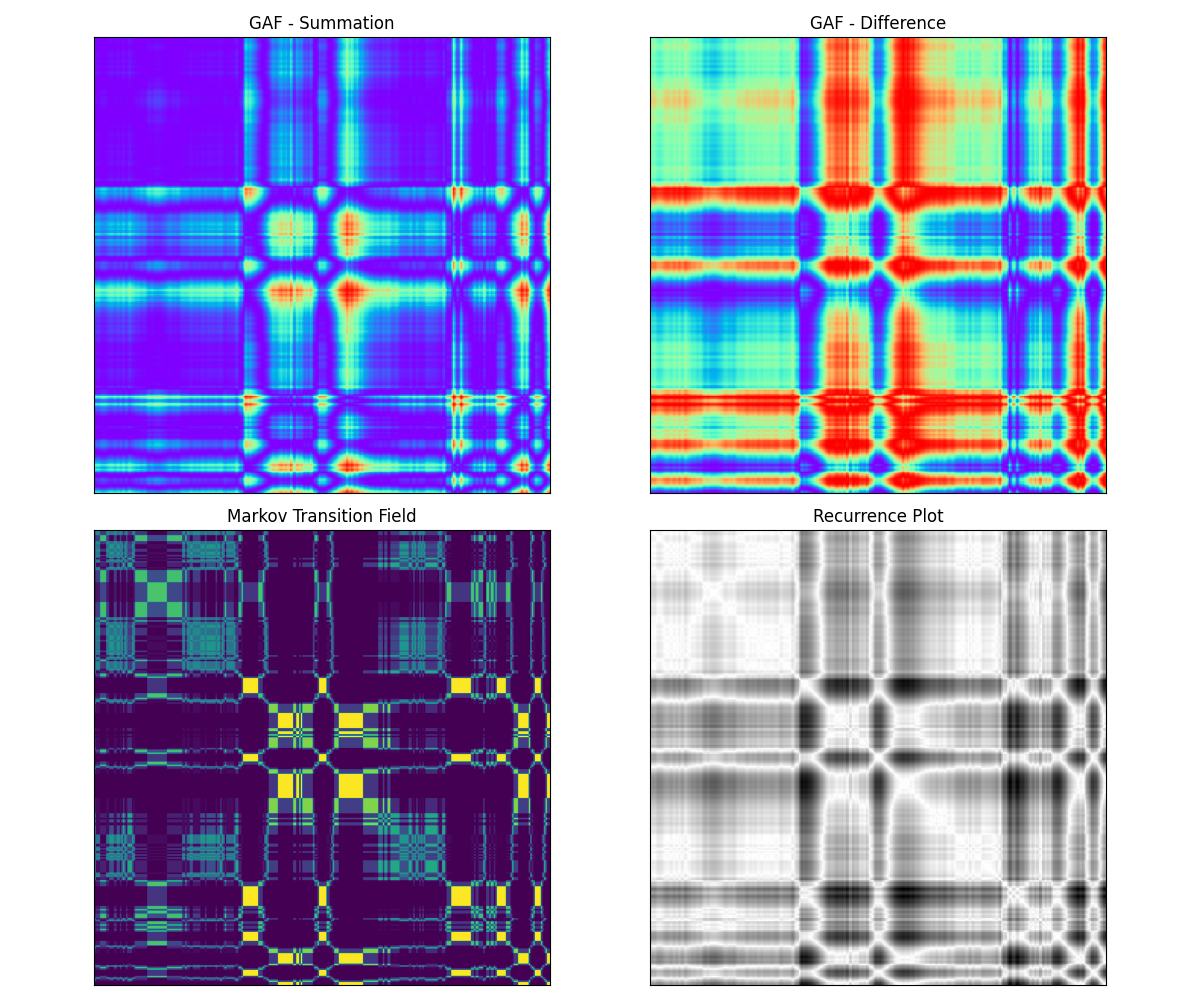


**Figure S8.** The Analysis for Choosing Markov Transition Field for EEG

The Markov Transition Field (MTF) offers significant advantages for the analysis of EEG signals by effectively capturing the temporal dynamics and probabilistic state transitions inherent in brain activity. Unlike methods such as Gramian Angular Fields (GAF), which emphasize symmetric correlations, or Recurrence Plots (RP), which focus on pairwise similarity, MTF encodes the probability of transitions between discretized signal states over time. This representation preserves both local and global temporal dependencies, making it particularly well-suited for EEG, where subtle changes in brain states occur continuously. Furthermore, MTF exhibits structured spatial patterns that can be readily exploited by convolutional neural networks (CNNs) for feature learning, thereby enhancing classification performance in tasks such as sleep stage detection, seizure prediction, or cognitive state recognition. Its reduced sensitivity to noise, combined with its ability to provide interpretable visual cues related to brain dynamics, makes MTF a robust and informative transformation for EEG-based research and applications.

***Mathematical Clarification Note:***

We define the multimodal feature fusion pipeline as follows:
Let

- $\mathbf{X}^{\mathrm{EEG}}\in\mathbb{R}^{n\times n}$ be the MTF-transformed EEG matrix,
- $\mathbf{X}^{\text{snore }}\in\mathbb{R}^{m\times k}$ be the MFCC-transformed snoring matrix,
- $f_{\text{res }}(\cdot)$ be the memristor reservoir response function applied to pulse-encoded inputs.

1. Reservoir Projection

Each modality is independently encoded as input pulse sequences and passed through the memristor-based physical reservoir:

$$\mathbf{R}^{\mathrm{EEG}}=f_{\text{res }}\left( \mathbf{X}^{\mathrm{EEG}} \right), \mathbf{R}^{\text{snore }}=f_{\text{res }}\left( \mathbf{X}^{\text{snore }} \right)$$

1. Feature Concatenation

The reservoir responses are flattened and concatenated:

$$\mathbf{F}=concat\left( \text{ flatten }\left( \mathbf{R}^{\mathrm{EEG}} \right),\text{ flatten }\left( \mathbf{R}^{\text{snore }} \right) \right)\in\mathbb{R}^{d}$$

1. Classification

The combined feature vector $\mathbf{F}$ is passed through a fully connected readout layer for inference:

$$y=\mathrm{Readout}(\mathbf{F})$$

This approach allows the fusion of heterogeneous modalities by leveraging the nonlinear transformation capacity of the physical reservoir, enhancing robustness and accuracy under noisy conditions.


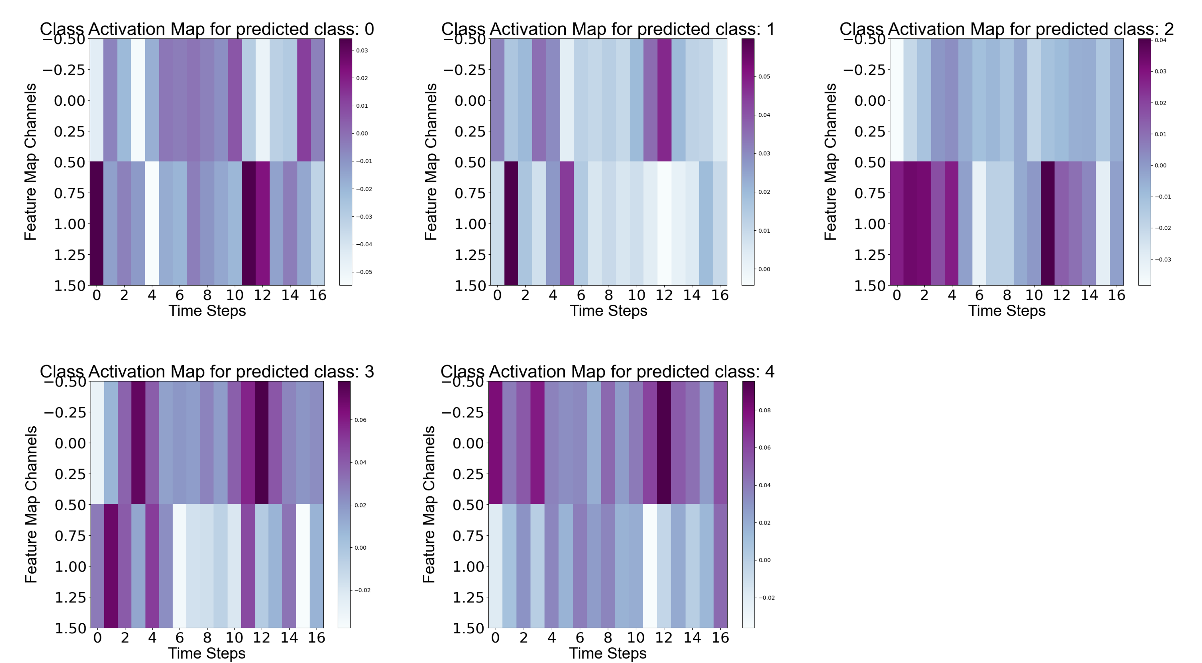


**Figure S9.** Class Activation Map of all 5 classes through the physical reservoir computing.


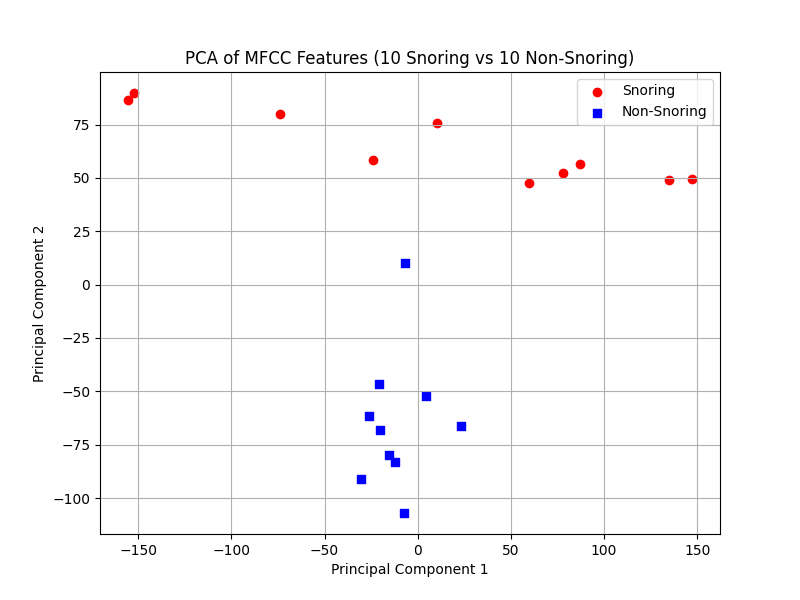


**Figure S10.** PCA for Snoring and non-Snoring in noisy background


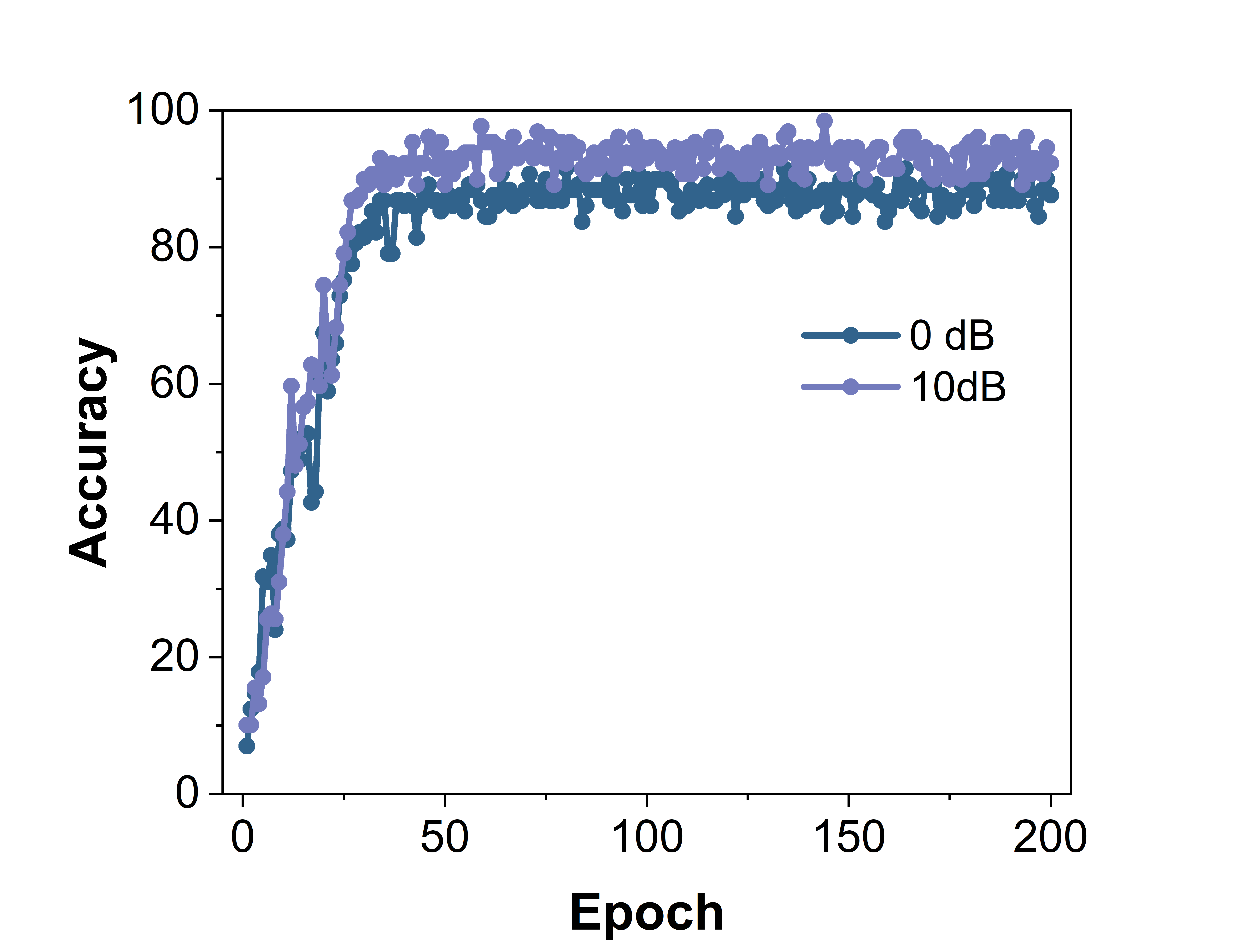


**Figure S11.** Robustness of multimodal classification under environmental noise.

Classification accuracy under different signal-to-noise ratios (SNRs), showing the impact of additive real-world noise (rainfall and traffic) on system performance. Accuracy remains high at 92.1% for 10 dB SNR and 88.3% for 0 dB SNR, confirming the system’s resilience under noisy conditions.


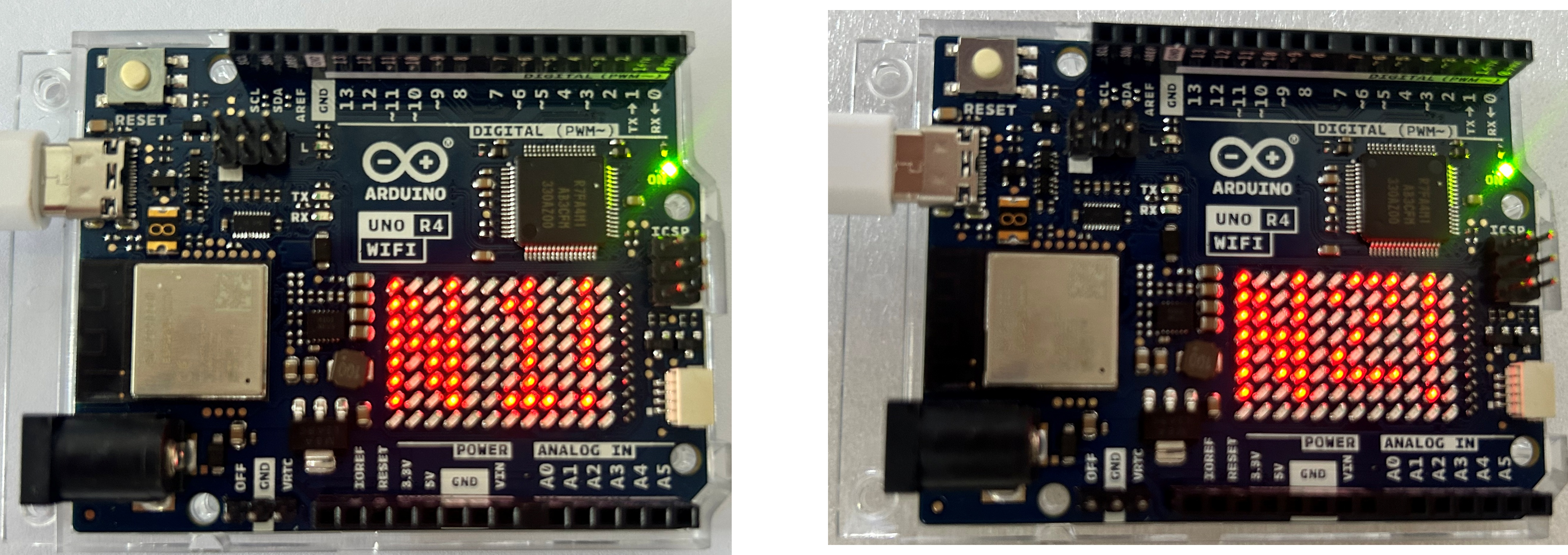


**Figure S12.**The Multimodal sleep detection result for different sleep stages with snoring


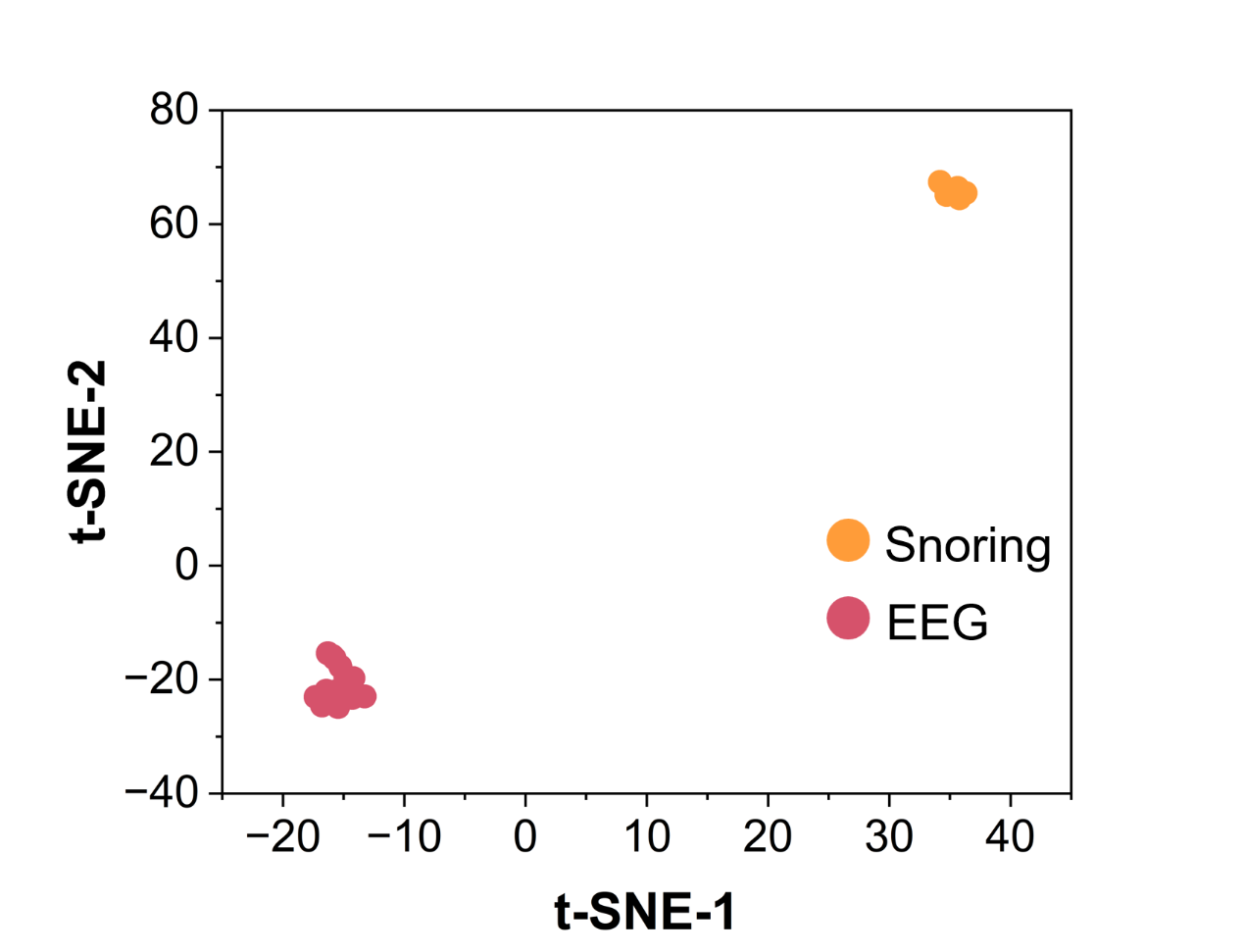


**Figure S13.**  t-SNE analysis for EEG and Soring signals


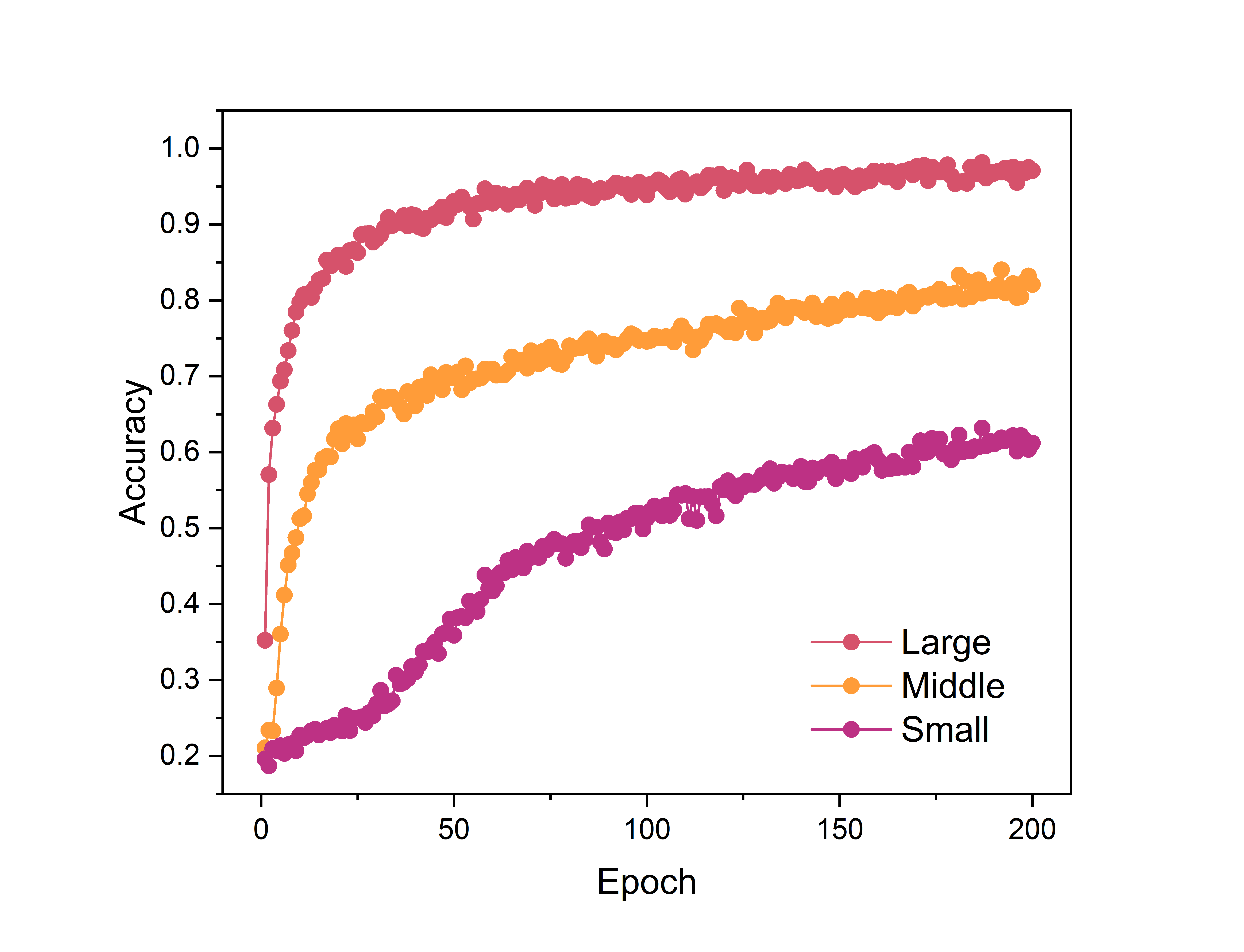


**Figure S14.** Accuracy of 3 scales for Physical Reservoir Computing.

**
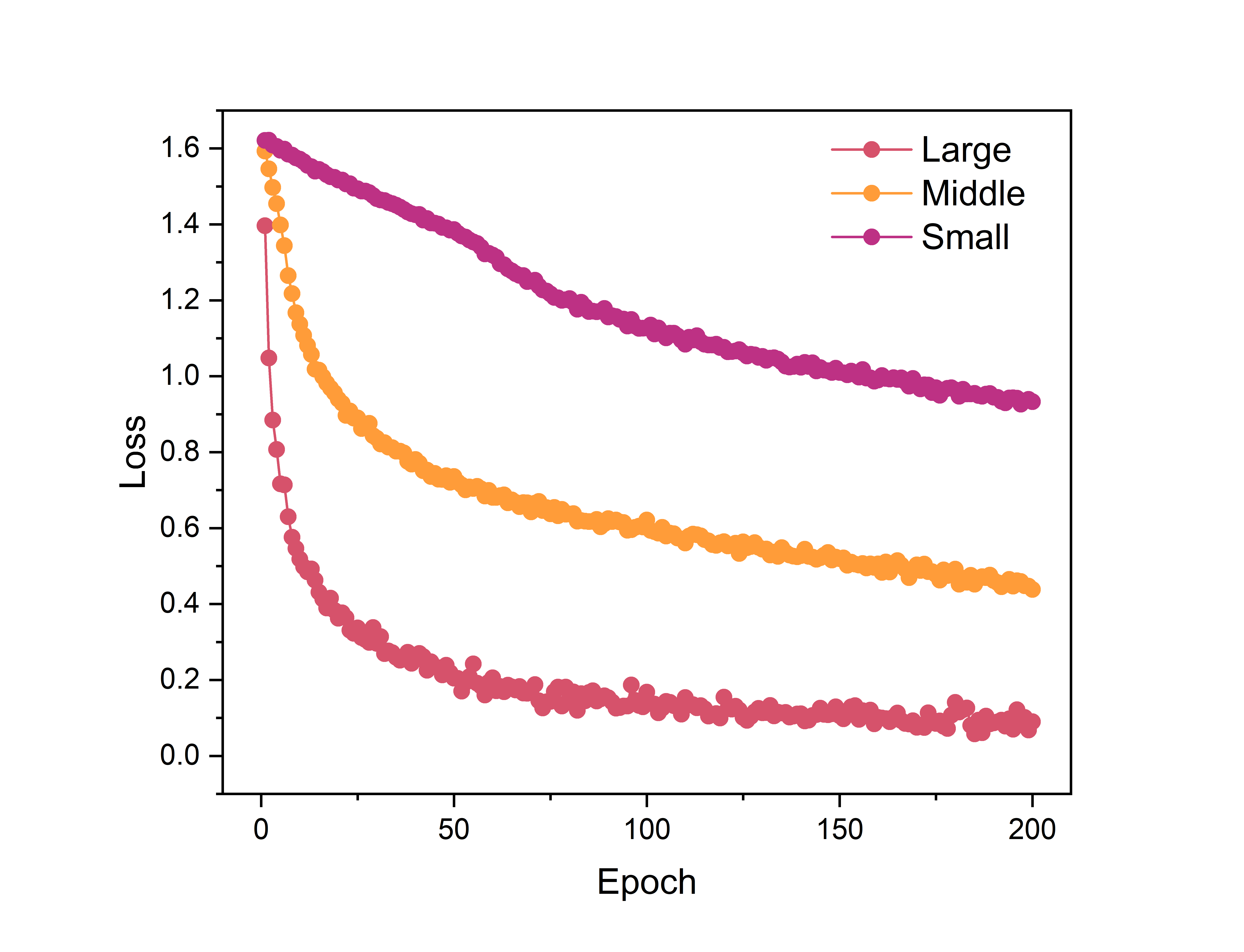
**

**Figure S15.** Loss of 3 scales for Physical Reservoir Computing.


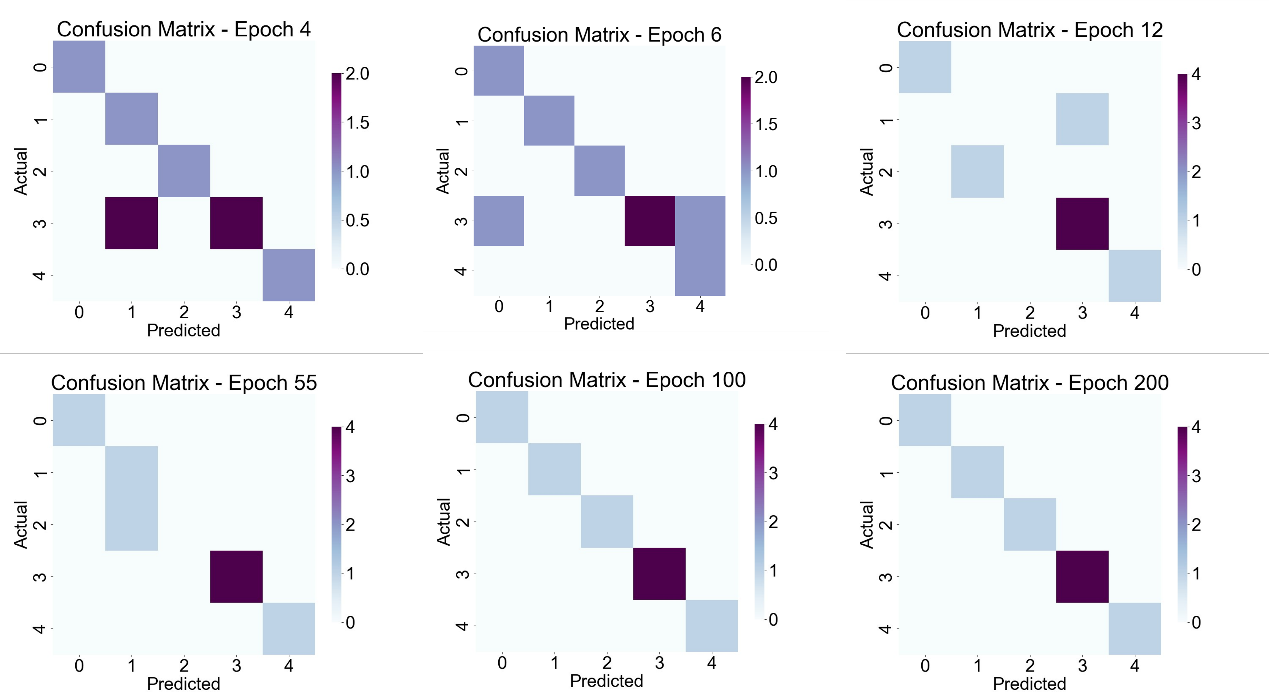


**Figure S16** Confusion Matrix for Physical Reservoir Computing.


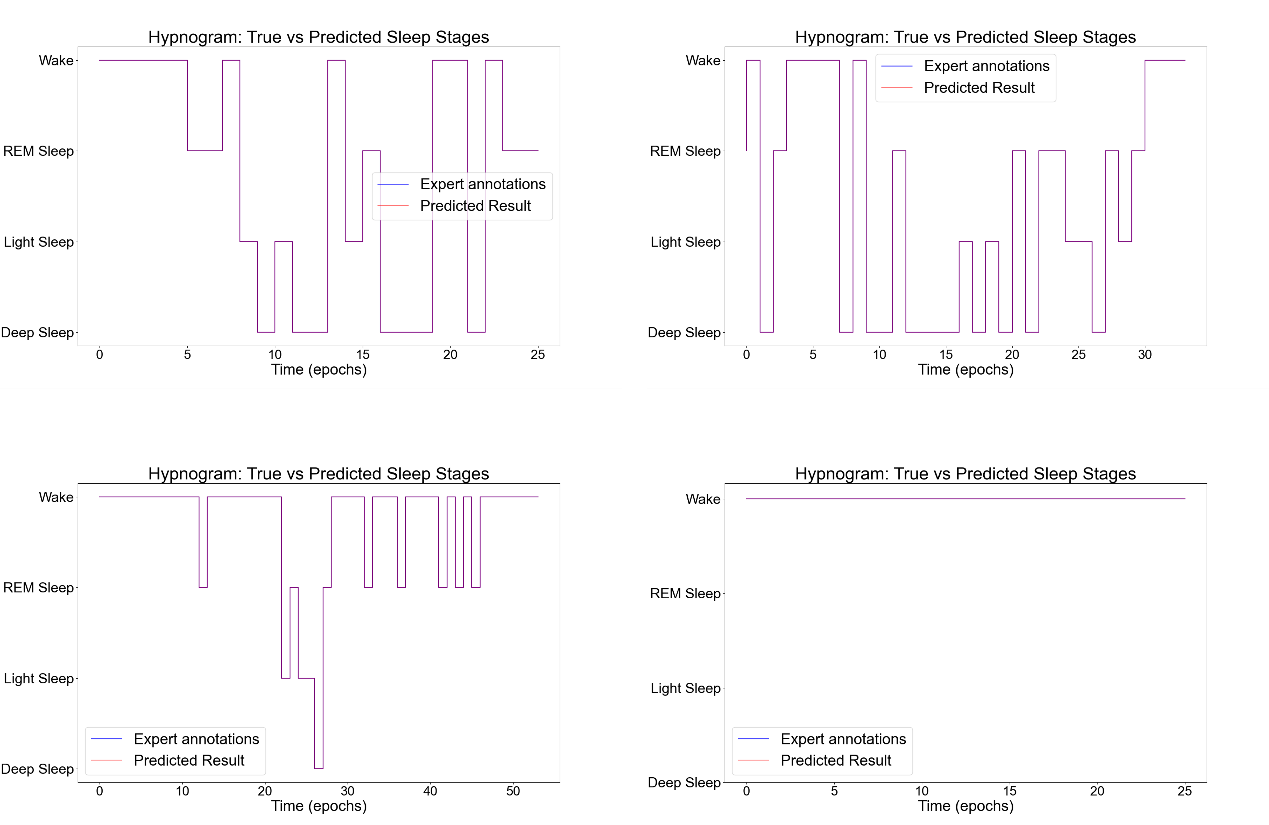


**Figure S17.** Hypnogram of True and Predicted Sleep Stages Results.

**
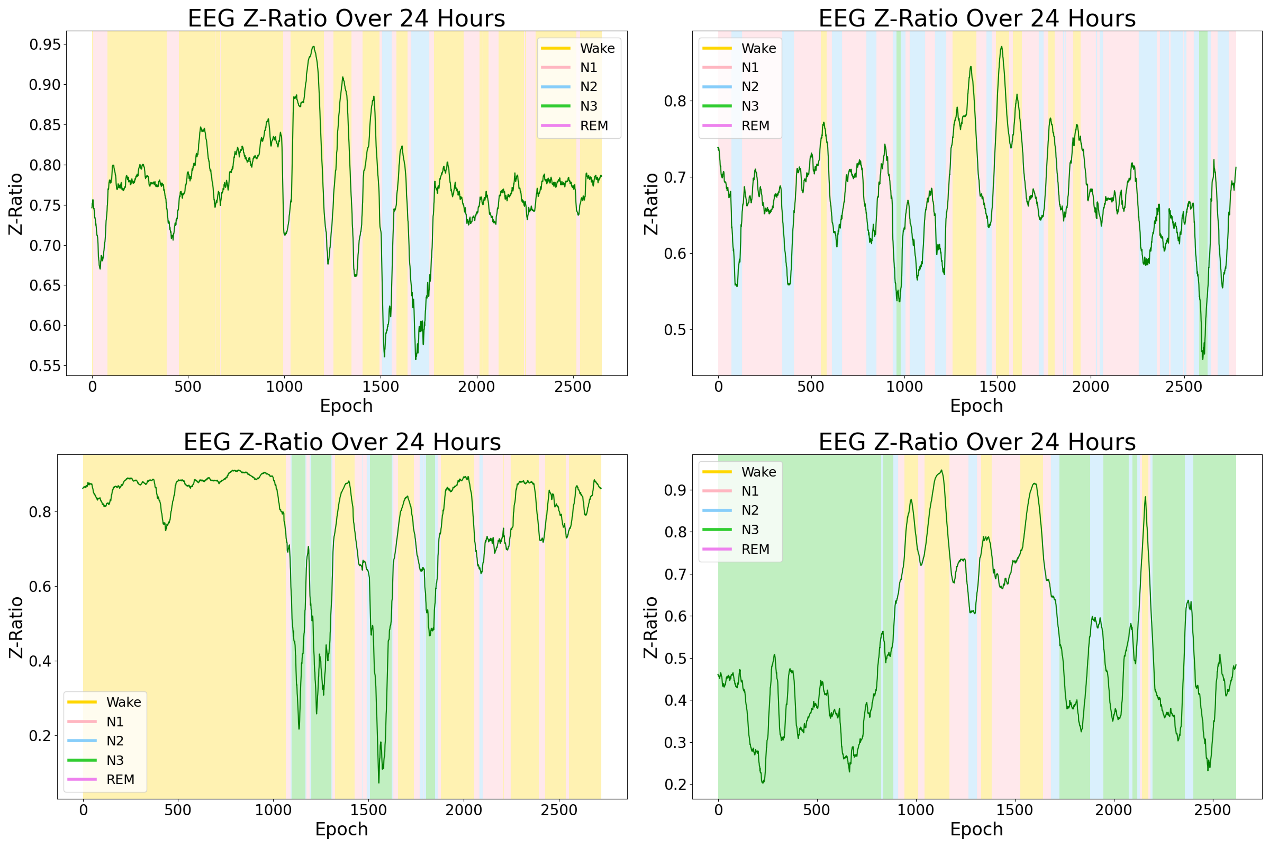
**

**Figure S18.** EEG Z-Ratio Over 24 Hours by physical reservoir computing.


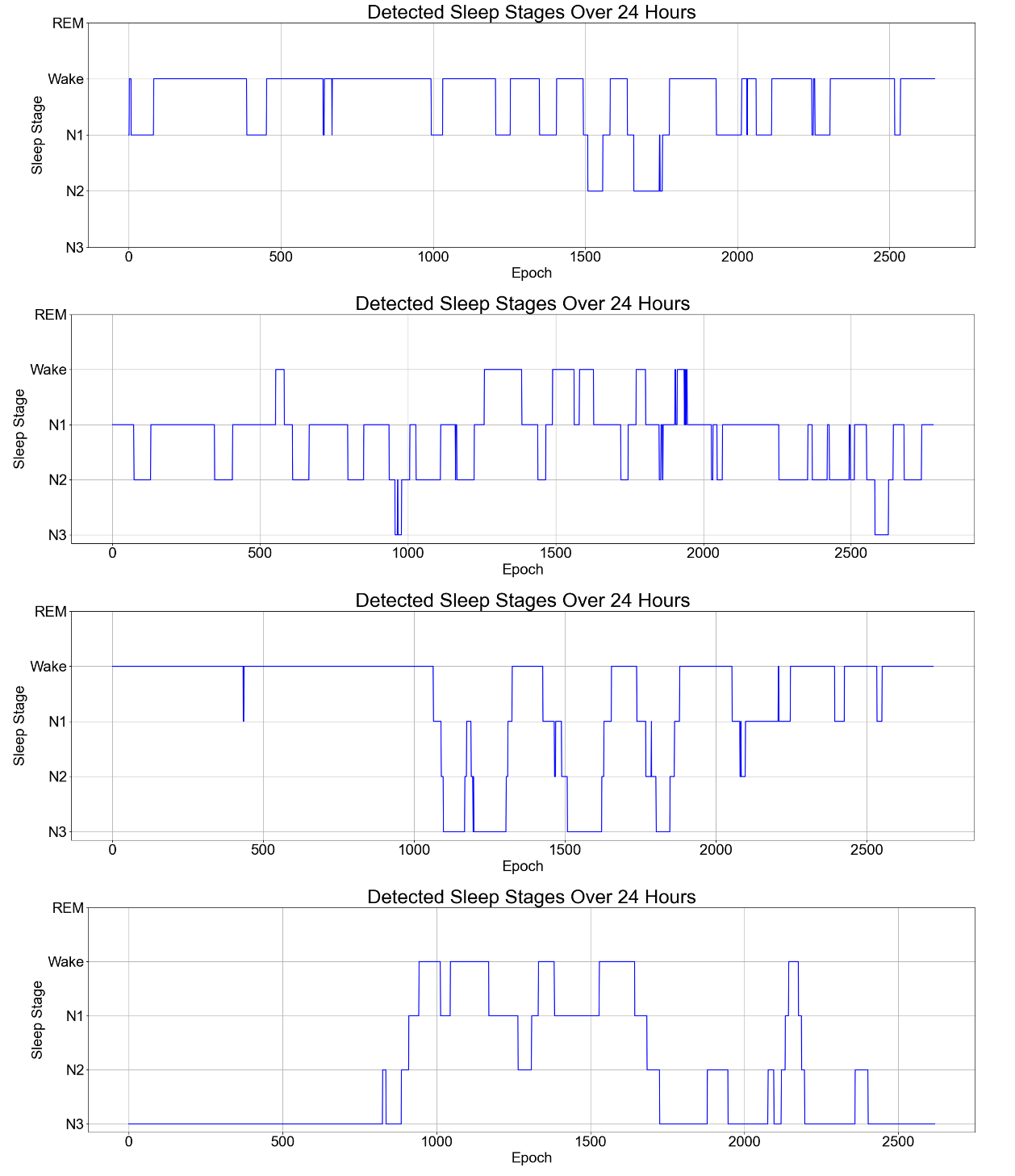


**Figure S19.** Detected Sleep Stages Over 24 Hours by physical reservoir computing


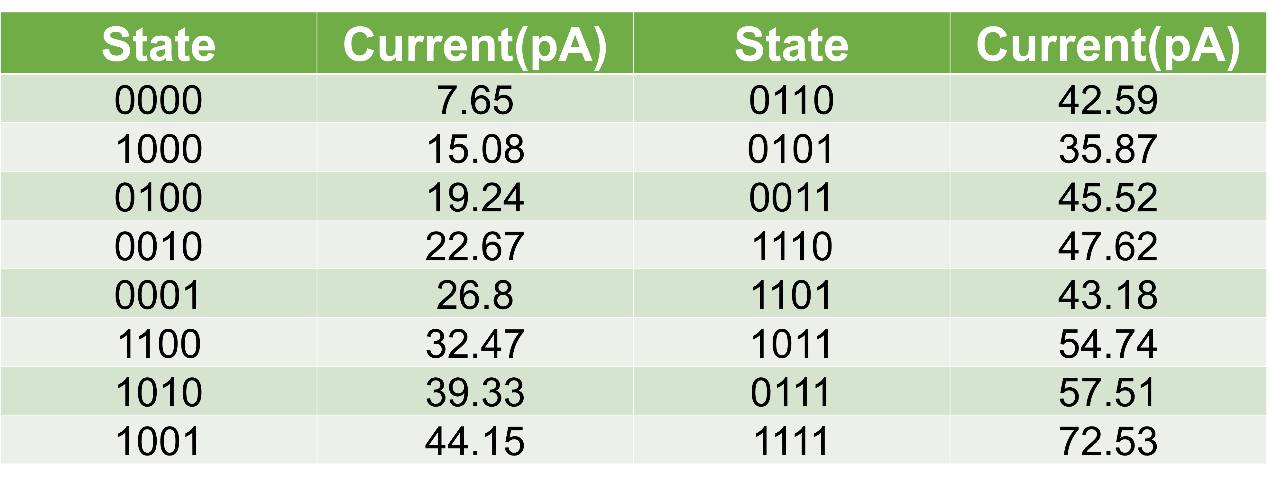


**Table S1.** Initial Experimental Final Current Data
